# Immune Checkpoint Inhibitors in Hepatocellular Carcinoma: Current Status and Novel Perspectives

**DOI:** 10.3390/cancers12103025

**Published:** 2020-10-18

**Authors:** Piera Federico, Angelica Petrillo, Pasqualina Giordano, Davide Bosso, Antonietta Fabbrocini, Margaret Ottaviano, Mario Rosanova, Antonia Silvestri, Andrea Tufo, Antonio Cozzolino, Bruno Daniele

**Affiliations:** 1 Medical Oncology Unit, Ospedale del Mare, 80147 Napoli, Italy; angelic.petrillo@gmail.com (A.P.); giopas@email.it (P.G.); davidebosso84@gmail.com (D.B.); antonietta.fabbrocini@gmail.com (A.F.); margaretottaviano@gmail.com (M.O.); rosanovamario@hotmail.com (M.R.); antonia.silv@libero.it (A.S.); b.daniele@libero.it (B.D.); 2 Division of Medical Oncology, Department of Precision Medicine, School of Medicine, University of Study of Campania “L. Vanvitelli”, 80131 Napoli, Italy; 3 Department of Clinical Medicine and Surgery, University of Naples “Federico II”, 80131 Naples, Italy; 4 Surgical Unit, Ospedale del Mare, 80147 Napoli, Italy; tufo.andrea@gmail.com; 5 Gastroenterology Unit, Ospedale del Mare, 80147 Napoli, Italy; ancozzolino@libero.it

**Keywords:** hepatocellular carcinoma, immunotherapy, combination therapy, predictive markers

## Abstract

**Simple Summary:**

Immune checkpoint inhibitors represent a promising treatment choice in many kind of tumours, including hepatocellular carcinoma (HCC). In this review, we provide an overview of the role of these new agents in the management of HCC according to the Barcelona staging system, alongside with a critical evaluation of the current status and future directions. Several clinical trials are focusing on the use of immunotherapy in HCC, alone or in combinations with antiangiogenetic agents as well as local treatment. However, the majority of those trials are still ongoing and, until now, only a few combinations were approved in the clinical practice from the regulatory authorities. Additionally, decisions about the choice of the right sequence of treatments in HCC patients in the light of the “continuum of care” principles, is still hard. In fact, it requires careful consideration in a multidisciplinary context in order to ensure a tailored treatment for each patient.

**Abstract:**

Immune checkpoint inhibitors (ICIs) represent a promising treatment for many kinds of cancers, including hepatocellular carcinoma (HCC). The rationale for using ICIs in HCC is based on the immunogenic background of hepatitis and cirrhosis and on the observation of high programmed death-ligand 1 (PD-L1) expression and tumor-infiltrating lymphocytes in this cancer. Promising data from phase I/II studies in advanced HCC, showing durable objective response rates (~20% in first- and second-line settings) and good safety profile, have led to phase III studies with ICIs as single agents or in combination therapy, both in first and second line setting. While the activity of immunotherapy agents as single agents seems to be limited to an “ill-defined” small subset of patients, the combination of the anti PD-L1 atezolizumab and anti-vascular endothelial growth factor bevacizumab revealed a benefit in the outcomes when compared to sorafenib in the first line. In addition, the activity and efficacy of the combinations between anti-PD-1/anti-PD-L1 antibody and other ICIs, tyrosine kinase inhibitors, or surgical and locoregional therapies, has also been investigated in clinical trials. In this review, we provide an overview of the role of ICIs in the management of HCC with a critical evaluation of the current status and future directions.

## 1. Introduction

Hepatocellular carcinoma (HCC) is the first primary liver cancer in incidence, showing 65,000 new cases/year in Europe, and the third cause of cancer related death worldwide [1]. The most important risk factors for HCC are chronic infections from B and C hepatitis virus (HBV and HCV, respectively), alcoholic cirrhosis, nonalcoholic fatty liver disease, aflatoxin B1, hemochromatosis, as well as other causes of cirrhosis [2].

Patients affected by HCC are complex and require a careful management in a multidisciplinary context involving experts in the field. In fact, they usually have concurrent diseases, such as cirrhosis or metabolic alterations, as well as history of alcohol abuse or liver interventions, which lead to a poor liver condition, eventually with portal hypertension and gastric and esophageal bleeding. Starting from this assumption, patients with HCC should be referred to dedicated centers and receive a multidisciplinary assessment at the diagnosis and during the entire treatment period. In this process, the staging evaluation is crucial in the HCC management algorithm in order to determine the outcomes and the treatment allocation. Among different staging systems, the Barcelona Clinic Liver Cancer (BCLC) is the more used, representing the accepted standard according to the study of Liver Disease (AASLD) and the European Association for the Study of the liver [3]. It combines multiple variables into an algorithm and identifies five stages for the disease: Patients with early HCC (stage 0/A) who are candidates for curative-intent radical therapies such as resection, liver transplantation and ablation; patients with multinodular tumours (stage B, intermediate) and candidate to local treatment, such as chemoembolization; those in advanced stage (stage C), eligible for systemic treatments and patients in terminal stage (stage D) for whom palliative cares are recommended. Switching to systemic therapy after locoregional treatment failure is known to have a crucial role in the decisions-making process in patients with trans-catheter arterial chemoembolization (TACE) refractory and intermediate stage HCC, since this transition has a great improvement impact on survival [4,5,6]. Therefore, clinicians should be careful in detecting the optimal timing for TACE refractory patients (TACE toxicity, disease progression after one or two courses of TACE, absence of a response, vascular or extrahepatic spread) to switch to systemic treatment [7].

Regarding stage C, according to international guidelines [8], sorafenib and lenvatinib represent the standard-of-care options in the first-line treatment. In patients who show a progression to first-line treatment, up to date, the multikinase inhibitors regorafenib and cabozantinib or ramucirumab, the anti-vascular-endothelial growth factor-2 (VEGF-R2), are the main choices in the second-line setting [9,10,11]. However, the small magnitude of survival benefit obtained with those tyrosine kinase inhibitors (TKI) and their poor tolerability have brought out the need for new therapeutic strategies.

In this context, the immune checkpoint inhibitors (ICIs) might represent the most important novelty and the future perspective also in the field of HCC. Indeed, over the last decades the understanding of the relationship between cancer and the immune system has progressed and ICIs have shown to improve the outcomes of patients in many kind of tumours, replacing the chemotherapy in some cases [12]. Regarding HCC, its peculiar immunogenic microenvironment has encouraged the use of immunotherapeutic agents, firstly revealing a potential role for pembrolizumab and nivolumab [13,14,15]. Recently, the regimen of atezolizumab and bevacizumab showed significantly longer overall survival (OS) and progression-free survival (PFS), as well as better patient-outcomes than sorafenib in the first-line systemic treatment [16]. These results identify not only the first therapy to improve survival beyond sorafenib over years, but also the first successful combination therapy and the first positive randomized phase III trial of ICIs in this challenging cancer. However, despite the advances in HCC treatment, only a small subset of patients respond to immunotherapy. Therefore, new tools to identify prognostic and predictive biomarkers able to select those patients who might actually benefit from ICIs -based treatment are urgently needed.

The aim of this review is to delineate an overview the biologic rationale for using immunotherapies in HCC according to BCLC stage, the current status and recent advances, alongside with the discussion of the areas for improvement and future implications.

## 2. Immunotherapies in HCC: The Biological Rationale of Their Use

Immunotherapy in HCC is particularly attractive for several reasons. The liver is an immunological organ that works as a biological filter against infections, which could came from the blood flow or gastrointestinal tract in which there might be a release of proteins and pro-inflammatory cytokines [17,18]. In fact, the liver is constantly exposed to many kind of antigens from food and microbiota, which can stimulate immune cells from innate and adaptive immune system. Several trials have demonstrated that the liver has developed an immune tolerability during its evolution process, due to the permanent exposure to those antigens [19,20]. This fact is supported by the evidence of the low rates of allograft rejection into the liver if compared to other organ transplants [21,22]. In addition, HCC is considered an inflammation-related cancer with a potential immunogenicity. In fact, it is know that the majority of HCC arises in liver affected by cirrhosis and hepatitis, which are considered typical inflammatory conditions [23]. Therefore, this inflammatory environment could act as “pro-neoplastic” factor, since it is involved in cancer progression through different mechanisms, such as the DNA damage and genomic aberrations.

**Figure 1:**
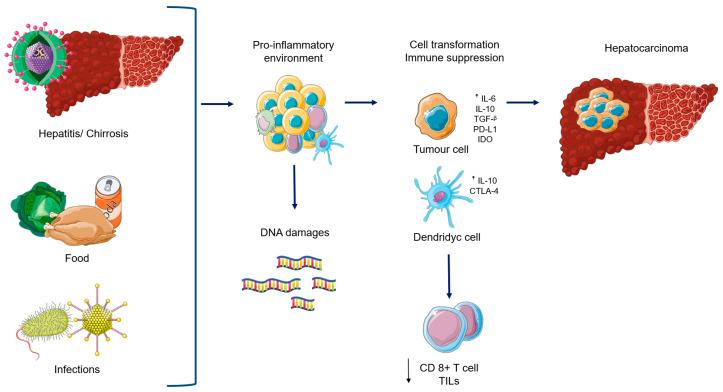
Biological rational to use Immune checkpoint inhibitors in Hepatocellular carcinoma. Abbreviations: IL-6: interleukin 6; IL-10: interleukin 10, TGF-β: tumor growth factor β PD-L!: programmed death ligand-1; IDO: Indoleamine Dioxygenaje, CTLA-4: Cytotoxic T-Lymphocyte Antigen 4; TILs: tumour infiltrating lymphocytes.

However, even if the liver has an “immunosuppressive” basal condition, several trials showed that an immune response to tumour is possible also in HCC. In particular, in patients who developed HCC after drug-induced immunosuppression, the discontinuation of immunosuppressive treatment lead to a spontaneous tumor regression by the reactivation of cytotoxic T-cells, that are able to identify and eliminate the cancer cells [24,25]; there is an increase in programmed death 1 (PD-1) and its ligand (PD-L1) expression as well as tumour infiltrating lymphocytes (TILs) in patients with HCC [26,27], leading to the immunosuppression [28,29]. Additionally, high Cytotoxic T-Lymphocyte Antigen 4 (CTLA-4) expression on regulatory T-cells (Tregs) in peripheral blood has been recorded in HCC patients in association with a decrease in immunosuppressive cytolitic granzyme B production by CD8+ T-cells. Regarding CD4+ T-cells, CTLA-4 is essential for their activation during the priming phase of the immune response. In fact, in the physiological process, T-cell are activated after the antigen presentation, whereas CTLA-4 reduces this activity, leading to T-cell suppression by blocking the binding between CD28 and CD80- CD86. Additionally, it plays an important role in the function of Tregs; in fact, CTLA-4 expression on CD14+ dendritic cells inhibits T-cell proliferation, inducing at the same time, the apoptosis of these cells by increasing the production of IL-10 and indoleamine-2,3-dioxygenase (IDO). Based on this background, there is a strong rationale to test ICIs in HCC [30] (Figure 1).

## 3. Immune Checkpoint Agents: Mechanism of Action and Their Use in HCC

Recently, the systemic management of HCC has been revolutionized by the advent of ICIs, a therapeutic class of monoclonal antibodies that blocks the immune checkpoints. These are co-inhibitory molecules physiologically expressed in different cells types, such as natural killer cells, dendritic cells, tumor-associated macrophages, monocytes and myeloid-derived suppressor cells (MDSCs)—including B and T cells, and that mantains self-tolerance [31,32]. ICIs act by applying a break that prevent the activation of these cells, limiting tissue damage. The balance between co-stimulatory signals and immune checkpoints leads to T cells activation, defining the intensity of the immune response.

ICIs have become a mainstay in the treatment of many cancers and then numerous clinical trials have been conducted and others are still ongoing with the aim to evaluate the safety and efficacy of these agents in several solid and hematological malignancies [12]. The main immune checkpoint receptors are CTLA-4, PD-1, TIM-3, BTLA, VISTA, LAG-3 and OX-40 [33].

The success of cancer immunotherapies through PD-1 and CTLA-4 mediated immunosuppression led to the developement of many clinical trials also in HCC. In this field, two classes of ICIs are currently being tested as mono or combination therapies: CTLA-4 (tremelimumab and ipilimumab) and PD-1/PD-L1 inhibitors (anti-PD-1: nivolumab, pembrolizumab, tislelizumab, camrelizumab and sintilimab; anti PD-L1: atezolizumab and durvalumab).

In the sections below we provide a short description of ICIs mechanism of actions, followed by the current state of advancement of ICIs-based therapies in HCC, according to BCLC stages.

### 3.1. The Checkpoint Pathways Regulated by CTLA-4 and by PD-1 Receptors

CTLA-4 is expressed on activated T cells, Tregs, and at low levels, on naïve T cells [34,35]. Its main function is to downregulate the activation of T lymphocytes by blocking the co-stimulatory signal of CD28 (for other details see Section 2).

PD-1 plays a key role in the regulation and in the maintenance of the balance between T cells activation and immune tolerance. Unlike CTLA-4, PD-1 is widely expressed, it can be detected not only on the T cell surface, especially CD8+ T cells, but also on Tregs and MDSC [36]. Additionally, whereas CTLA-4 mainly regulates the activation of T cells in lymphatic tissues, the most important action of PD-1 is to reduce the activity of T cells in peripheral tissues during the immune cell mediated or inflammatory response. Then, T cell function is influenced by the level of PD-1 activity and, when PD-1 binds to its ligands PD-L1 or PD-L2, T cell proliferation and cytokine release are inhibited.

Today, it is well-know that chronic exposure to antigens leads to the hyperexpression of PD-1 in T cells [37]. Additionally, cancer cells can turn PD-L1/PD-1 signaling to their own advantage through the expression of PD-L1 or PD-L2. This can activate PD-1 in TILs, resulting in the escaper of immune surveillance [38,39]. The hyperexpression of PD-1 reported on CD8+ T-cells in patients with HCC and the increase in PD-1+CD8+ TILs confirmed the previous theory. Additionally, the presence of those cells in HCC specimens was associated with higher rate of progression of disease after curative hepatic resection [26,27].

### 3.2. ICIs-Therapies in HCC Patients According to BCLC Stage

#### 3.2.1. Early Stage HCC (BCLC stage 0 or A)

According to international guidelines [8], liver resection or ablation treatments are the standard of care for patients with BCLC stage 0 or A. Single tumors in patients with well-preserved liver function and no clinically significant portal hypertension [40] is the mainstay indication for resection, providing a survival rate of almost 60% at 5 years and no postoperative liver failure (postoperative mortality <3%). However, after liver resection, tumour recurrence can be observed in 50–70% of cases in 5 years, the majority in the first two years, and no adjuvant therapies have been shown to reduce recurrence rate in this field [5,41,42].

Ablation is preferred in patients with BCLC stage 0 or A, who are not candidates for surgery [8]. The main procedure is by percutaneous radiofrequency (RFA), which acts by causing ischemic cell damage with the release of neoantigens and promotion of immunogenic cell death [43,44].

**Figure 2:**
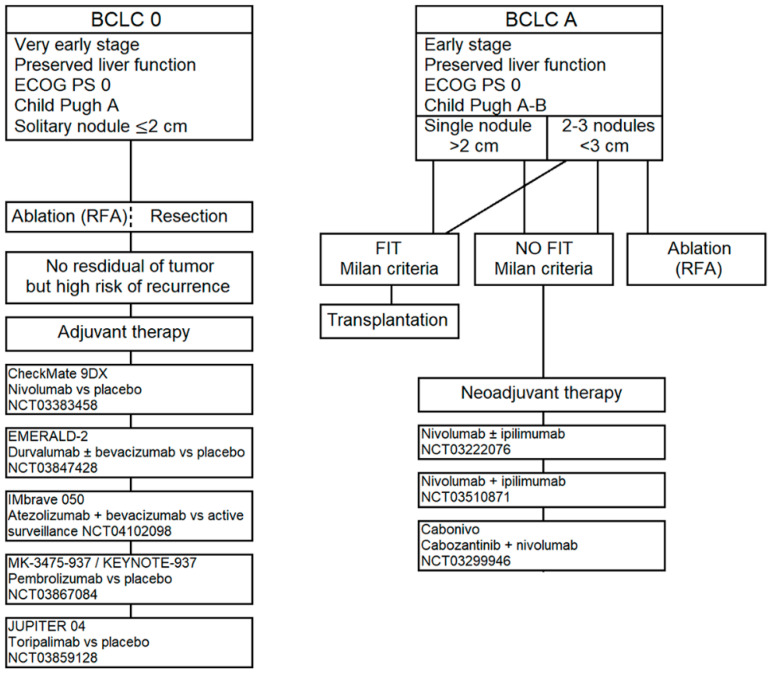
Treatment options and ICIs ongoing clinical trials for BCLC stage 0-A HCC.

Due to these considerations, ICIs have been thought to be beneficial in the adjuvant setting for patients with high risk of recurrence after complete resection or complete response by local ablation. Therefore, several clinical trials are ongoing in this regard. Figure 2 summarizes the treatment options for BCLC stage 0/A HCC and the ICIs ongoing clinical trials in this field.

In particular, the phase III, placebo-controlled CheckMate 9Dx trial (Clinical Trials.gov Identifier: NCT03383458) is investigating the role of nivolumab in this setting; the phase III EMERALD-2 trial (NCT03847428) is assessing the efficacy and safety of durvalumab as monotherapy or in combination with bevacizumab versus placebo; the phase III IMbrave050 (NCT04102098) is assessing the efficacy of atezolizumab plus bevacizumab versus active surveillance. Additionally, the safety and efficacy of pembrolizumab versus placebo as adjuvant therapy are being studied in the phase III trial KEYNOTE-937 (NCT03867084); the JUPITER-04 clinical trial (NCT03859128) is investigating the possible role of toripalimab (Recombinant Humanized Anti-PD-1 Monoclonal Antibody, JS001) in the improvement of relapse free survival (RFS) compared to placebo in the adjuvant treatment for patients who underwent complete liver resection, but with high risk of relapse.

It is worth remembering that liver transplantation can also be performed in patients with a limited tumor burden and fit the Milan criteria [45]. In these patients <10% recurrence rate and 70% five-year survival rate are expected [46]. However, the low availability of liver allografts is the major limitation for liver transplantation. In patients who do not fit the Milano criteria and in patients who are in the waiting list for transplant, the UNOS (United Network for Organ Sharing) allows the use of neoadjuvant treatments generally ablation or transarterial therapies; this is due to the long waiting period and the risk of tumor progression [8].

In the context of neoadjuvant setting for resectable HCC, the use of ICIs is currently being studied in several trials. In particular, the phase II randomized NCT03222076 trial is evaluating the safety and tolerability of nivolumab alone or in combination with ipilimumab; the interim analysis involving 8 patients showed a 37.5% of pathological complete response in the entire population with a good safety profile [47]. Additionally, the phase II NCT03510871 is evaluating the efficacy, in terms of tumor shrinkage, objective response rate and neoadjuvant down-stage rate, of nivolumab plus ipilimumab in this setting. Then, the phase I CaboNivo trial (NCT03299946) is assessing the feasibility and efficacy of cabozantinib plus nivolumab followed by definitive resection.

In conclusion, up to date, the treatment algorithm for patients with BCLC 0 or A HCC is unchanged and doesn’t include the use of ICIs. Further evaluations, as well as the results of those ongoing trials are awaiting in order to eventually improve the treatment’s choices.

#### 3.2.2. Intermediate—Stage HCC (BCLC Stage B)

Patients with intermediate-stage tumors should be considered for TACE, according to the current guidelines indications [8]. However, all the studies that have investigated the combination of sorafenib and TACE over the last decades did not reveal to improve the OS when compared with sorafenib or TACE alone [48,49]. For example, the TACTICS study, which evaluated the efficacy of TACE plus sorafenib versus TACE in unresectable HCC, showed to only improve PFS (25.2 versus 13.5 months, *p* = 0.006) [50].

**Figure 3:**
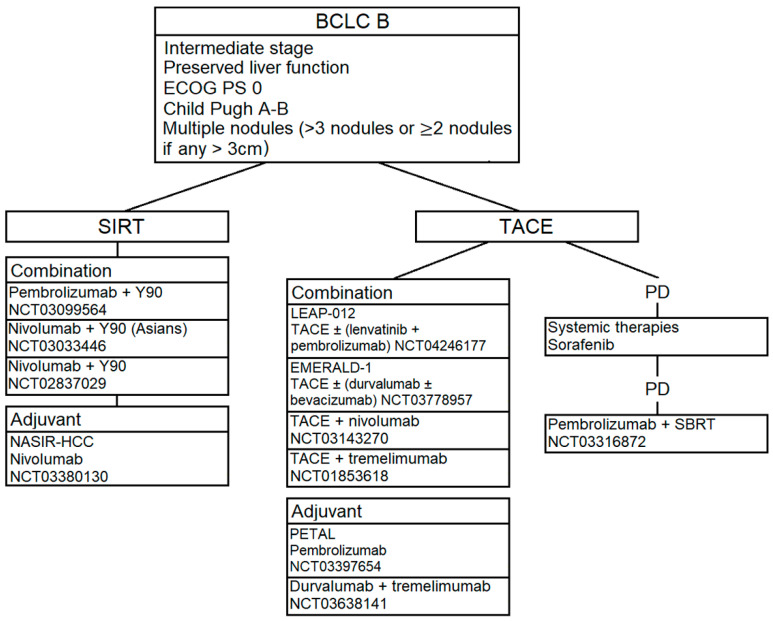
Treatment options and ICIs ongoing clinical trials for BCLC stage B.

In this context, there are newly evidences supporting the use of immunotherapy in the BCLC B stage, basing on the concept that a combination of ICIs and TACE may improve the efficacy of the standard treatment. Indeed, TACE leads to tumor necrosis and cellular damage by inducing high intratumoral temperature. This mechanism of action is responsible for the higher release of neoantigens, which promote an immunogenic environment [51]. Figure 3 summarizes the treatment options for BCLC stage B HCC and the ICIs ongoing clinical trials in this field.

Preliminary results of the phase I/II PETAL clinical trial, which evaluated the safety and activity of pembrolizumab after TACE, revealed a good tolerability for the sequential treatment without cumulative side effects [52]. Tremelimumab is also being evaluated with TACE/RFA in the ongoing NCT01853618 trial. Additionally, clinical trials testing TACE plus nivolumab (NCT03143270) and durvalumab plus tremelimumab following TACE (NCT03638141) are currently running.

Then, some studies are investigating the synergistic effects of different mechanisms of action in order to improve the patients’ outcome in this staging group. In fact, the ischemic cell damage related to TACE might produce an increase in the vascular endothelial growth factors (VEGFs) levels in addition to the increase in the immunogenic cell death and stimulation of a peripheral immune response. Therefore, the follow therapeutic combinations are being tested in this field: TACE plus pembrolizumab and lenvatinib (LEAP-012 trial, NCT04246177); TACE plus durvalumab and bevacizumab (EMERALD-1 study, NCT03778957).

Selective internal radiation therapy (SIRT) is another transaerterial approach used in patients with BCLC stage B tumors. It consists in the intraarterial infusion of microspheres with the radioisotope Yttrium-90. According to retrospective studies, SIRT determines objective responses similar to TACE [53]; however, no data about survival are available in this field, due to the lack of phase III comparative studies between TACE and SIRT. Regarding ICIs, several trials in combination with SIRT are currently recruiting. NCT03099564 is an open-label multi-center trial assessing the efficacy and safety of pembrolizumab with Yttrium-90; NCT03033446 trial is a phase II study with the objective is to evaluate the effect of SIRT in combination with nivolumab in Asian patients, also pre-treated with prior local therapies. Additionally, the phase I NCT02837029 trial is identifying the maximum tolerated dose of nivolumab for combination treatment of nivolumab and Yttrium-90. Then, NASIR-HCC study (NCT03380130) completed the enrollment with the aim to evaluate the safety and the antitumoral efficacy of nivolumab after SIRT for patients with unresectable HCC, who were candidates for locoregional therapies.

At least, in patients with BCLC stage B who progressed to transarterial therapies, a systemic treatment is recommended instead of multiple local therapies [8]. After progression to sorafenib, the phase II NCT03316872 study is testing the efficacy of the combination of pembrolizumab and stereotactic body radiotherapy (SBRT).

#### 3.2.3. Advanced—Stage HCC (BCLC stage C)

**Figure 4:**
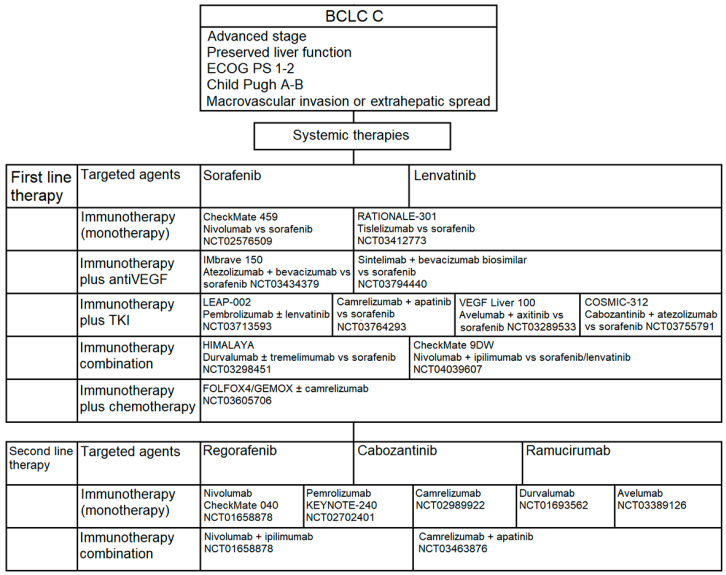
Treatment options and ICIs ongoing clinical trials for BCLC stage C.

Systemic therapies are indicated in patients with advanced disease (BCLC stage C) or intermediate stage disease (BCLC stage B), who are not eligible for locoregional therapies or after progression to local treatment, as already mentioned. According to international guidelines [8], target therapy with TKIs is the standard of care in the first line treatment, whereas there is no indication to use chemotherapy in this setting due to the lack of efficacy. In this context, immunotherapy represents an exciting treatment alternative to explore (Figure 4).

##### First-Line Therapy: From the Standard of Care to the New Frontiers

Historically, Sorafenib was the first systemic drug approved by Food and Drug Administration (FDA) for the treatment of advanced HCC and has remained a unique and effective standard of care for frontline therapy for approximately 10 years [54]. In 2018, Lenvatinib (another TKI) received the FDA approval for advanced HCC on the basis of the phase III non-inferiority REFLECT trial, which excluded conditions with the main portal vein invasion, clear bile duct invasion and >50% of tumour total liver volume occupancy [9]. Based on this background, immunotherapy was considered a promising alternative to treatment with TKI. Therefore, the number of trials evaluating the role of ICIs in the first line therapy for advanced HCC has increased, both in monotherapy and in combination with other ICIs or targeted/antiangiogenetic agents.

More in details, the phase III CheckMate-459 trial evaluated the efficacy and safety of nivolumab (a fully human anti-PD-1 IgG4 antibody administrated at 240 mg every two weeks) versus sorafenib as first line therapy in patients with unresectable HCC. The study did not reached its primary endpoint (OS: 16.4 versus 14.7 months in the experimental and standard arm, respectively; Hazard Ratio (HR): 0.85; 95% Confidence Interval (CI): 0.72–1.02; *p* = 0.0752) [55]. ORR were 15% and 7% in the nivolumab and sorafenib arm, respectively; a clinical benefit was reported in all the pre-planned subgroups, including those according to hepatitis infection status, presence of vascular invasion and/or extrahepatic spread, geographical region (Asia versus non-Asia). Of note, 140 patients (38%) in the experimental arm and 170 patients (46%) in the control arm received subsequent lines of treatment. Though the primary endpoint was not met, nivolumab showed clinically meaningful improvements in OS, ORR and complete response rate as well as favorable safety profile as first line treatment.

Tislelizumab (BGB-A317) is another humanized IgG4 antibody against PD-1 tested in the front-line treatment for advanced HCC. Based on promising results of the phase I trial, involving 61 patients with solid cancers included HCC [56], the phase III non-inferiority RATIONALE-301 trial compared tislelizumab (200 mg every three weeks) versus sorafenib [57]. The trial is currently ongoing (NCT03412773) with an expected end date on June 2021.

Despite interesting results from ICIs monotherapy studies in HCC, only a small group of patients benefits from ICIs [58]. Thus, several combination approaches have been utilized with the aim to improve the anti-tumor efficacy and survival in the whole HCC population, targeting different pathways. Well-known combinations included: anti PD-/PD-L1 antibody with non-immune-based-therapies (TKI, anti-VEGF, chemotherapies); two types of ICIs (anti PD-1/PD-L1 and anti CTLA-4 antibodies); ICIs with existing locoregional therapies (already discussed above; see Section 3.2.1 and Section 3.2.2).

Atezolizumab plus bevacizumab is one of the most interesting combination tested in this field. The rationale of the combination could be found in preclinical studies, in which bevacizumab showed to enhance PD1/PD-L1 efficacy by reversing VEGF-mediated immunosuppression and by promoting tumor T-cell infiltration [59]. Indeed, during the process of carcinogenesis, the VEGF stimulates the formation of new vessels (angiogenesis), reducing simultaneously the immune response against the tumor. Therefore, the use of anti-VEGF drugs could have a double effect on cancer cells, which is antiangiogenic and immunomodulation. More in details, the VEGF would exercise its immunosuppressive role through three main ways: reducing T cell activation by inhibition of the maturation of dendritic cells through nuclear factor kB; creating aberrant tumor vessels and down-regulating the selectins and adhesion molecules (necessary for the adhesion of T cells to the vascular endothelium itself); increasing the number of inhibitory immune cells in the tumor microenvironment. In this light, bevacizumab might improve the tumour immunogenity, leading to a stronger host immune-response.

In July 2018, the FDA assigned the breakthrough therapy approval to atezolizumab in combination with bevacizumab in advanced HCC on the basis of the results of the phase Ib GO30140 Study [60]. The interim data analysis of this trial showed a response rate in 32% of patients by RECIST criteria. Responses were durable (≥6 months: 52%, ≥12 months: 26%), grade 3–4 treatment related adverse events (TRAEs) occurred in 27% of patients and hypertension was the most common (10%). Even if 2% of patients had a drug-related death, the combination was well-tolerated, having a good safety profile. Then, the phase III IMbrave150 study randomized 501 patients with unresectable or metastatic HCC, naïve to systemic therapy, to receive atezolizumab (1200 mg every three weeks) and bevacizumab (15 mg/kg every three weeks, *n* = 336 patients) or sorafenib (165 patients) [61]. The two primary endpoints were OS and independent review facility–assessed PFS per RECIST 1.1. The trial showed 42% OS improvement in the atezolizumab plus bevacizumab arm (HR= 0.58; 95% CI: 0.42–0.79, *p* = 0.0006) as well as 41% improvement in PFS (HR = 0.59; 95% CI: 0.47–0.76, *p* < 0.0001) if compared with sorafenib. It is noteworthy that, over the last decades, IMbrave150 is the first phase III positive trial, showing an improvement in both OS and PFS in this setting of disease for a new combination of drugs beyond sorafenib. Regarding the safety profile, 38% of patients had a grade 3–4 TRAEs in the combination arm; the most frequent were bleeding in the gastrointestinal tract, infections and fever [62]. The combination therapy also resulted in better quality of life outcomes (longer time to deterioration of quality of life and functioning) than treatment with sorafenib. Time to deterioration, that was the reduction of 10 points from the baseline-reported score, was 11.2 months in patients receiving atezolizumab and bevacizumab and 3.6 months in those treated with sorafenib (HR = 0.63; 95% CI: 0.46–0.85). Declines in physical and role functioning also improved in the experimental arm. Further, the physical functioning had a median delay of 13.1 versus 4.9 months for the experimental and control arm, respectively (HR = 0.53; 95% CI: 0.39–0.73), as well as the role functioning (median delay of 9.2 versus 3.6 months, respectively (HR = 0.62; 95% CI: 0.46–0.84)). Additionally, atezolizumab and bevacizumab delayed the time to deterioration. Combination treated patients reported appetite loss, fatigue, pain, and diarrhea in a lower proportion than sorafenib, experiencing less clinically meaningful deterioration in each of these symptoms. Based on those results, on January 2020 a supplemental Biologics License Application was submitted to the FDA for atezolizumab plus bevacizumab combination in the first-line treatment for advanced HCC [63]. The combination was finally approved by FDA in this setting [64].

Regarding other combinations between anti-PD-1 and anti-VEGF, the phase II/III ORIENT-32 trial (NCT03794440) is assessing the safety, tolerability and effectiveness of sintilimab in combination with IBI305 (anti-VEGF monoclonal antibody, bevacizumab biosimilar) in patients with HCC as the first-line treatment compared with sorafenib (estimated end date: December 2022).

Another intriguing combination is between ICIs and TKI. The rationale for their combination comes from the evidence that antiangiogenic mechanisms may increase tumor hypoxia, leading to the upregulation of the costimulatory molecule OX40 in T-cell-mediated immunity; OX40 promotes the survival and expansion of CD8+T cells and the recall response of CD8+memory T cells [65]. Examples of combinations of ICIs and molecular targeted therapy are: pembrolizumab plus lenvatinib; camrelizumab plus apatinib; avelumab plus axitinib; atezolizumab plus cabozantinib.

Starting from the first combination, the Keynote-524 is an open-label, phase Ib study which tested the safety of pembrolizumab and lenvatinib in patients with unresectable HCC, not amenable to locoregional treatments [66]. The trial had a safety lead-in of six patients with a subsequent expansion cohort of 24 previously untreated patients. It revealed ORR by RECIST and modified RECIST (mRECIST) of 36.7%, and 50%, respectively. Based on these promising preliminary results, the trial is actually involving 104 patients in the phase II and led to breakthrough FDA approval of the combination on July 2019. The phase III study LEAP-002 study (NCT03713593) is now evaluating lenvatinib as single agent or in association with pembrolizumab in the first line setting [67].

Regarding camrelizumab and apatinib, a phase III clinical trial is currently testing this combination versus sorafenib as first-line therapy in patients with advanced HCC (NCT03764293).

The phase Ib VEGF Liver 100 study (NCT03289533) investigated the safety of avelumab (10 mg/kg every two weeks) co-administered with the TKI axitinib (5 mg orally twice a day) as first line. The treatment was active, showing an ORR of 13.6% and 31.8% based on RECIST 1.1 or mRECIST criteria, respectively; median PFS was 5.5 and 3.8 months, based on RECIST and mRECIST, respectively. The study reported higher grade 3 TKI-TRAEs, especially hypertension (50%) and hand–foot syndrome (22.7%), in the experimental arm, but without grade ≥3 immune-related adverse events [68].

The phase III COSMIC-312 trial (NCT03755791) is comparing the association between cabozantinib and atezolizumab versus sorafenib in patients with advanced HCC naïve to systemic treatments.

The combinations between anti-PD-1 plus chemotherapy and anti-PD-L1 and anti-CTLA-4 represent the last most important combinations investigated in the field of first line treatment for advanced HCC. In the first case, the combination of camrelizumab plus FOLFOX-4 (5-fluorouracil plus oxaliplatin) or GEMOX (gemcitabine plus oxaliplatin) was tested in a phase II study, involving 34 patients [69]. The trial showed an ORR of 26.5%, a disease control rate (DCR) of 79.4% and a median PFS of 5.5 months. These data have led to investigate camrelizumab in combination with FOLFOX-4 in a phase III study (NCT03605706) in the same setting; the trial is currently running.

Regarding the combinations between anti PD-L1 and anti-CTLA-4, the rationale for their use consists in the ability of improving the immune stimulation by targeting different pathways; this strategy has been already investigated in many kinds of tumors with positive results [70]. In this subgroup the most important combinations are durvalumab plus tremelimumab (NCT03298451) and nivolumab plus ipilimumab (NCT01658878, NCT03222076, NCT03510871), both under investigation at the time of writing.

Durvalumab and tremelimumab have been investigated in a phase I/II study involving 40 patients with advanced HCC [71]. The trial used a schedule of durvalumab at the dose of 20 mg/kg and tremelimumab at the dose of 1 mg/kg every 4 weeks, followed by 20 mg/kg durvalumab as maintenance. In this study, it is important to note that 70% of the patients had received previous systemic therapies and half of the study population had no history of hepatitis. The ORR was 15% (all the responses were seen in patients without history of hepatitis); 16-weeks disease-control rate was 57%. However, there was 20% of serious TRAEs, leading to the discontinuation of treatment for toxicity in 7% of patients. Based on these results, the randomized phase III HIMALAYA study (NCT03298451) is currently running. The trial is investigating the efficacy and safety of durvalumab plus tremelimumab or durvalumab as single agent versus sorafenib as first-line treatment for patients with naïve unresectable HCC. The preliminary safety results showed that the combination was well tolerated [72]; the most common all-grade TRAEs included fatigue (27.5%), increased alanine aminotransferase (ALT; 20.0%), pruritus (22.5%), increased aspartate aminotransferase (AST; 17.5%), elevated lipase (10.0%). Twenty-five percent of patients experienced grade 3/4 TRAEs or serious AEs and no treatment-related deaths occurred. Of note, this trial represents the first phase III study that have evaluated a combination between two ICIs as first-line treatment for advanced HCC. Therefore, in January 2020, the FDA approved the combination of durvalumab and tremelimumab in this field, designing these agents as orphan drugs [73].

Last, the phase III CheckMate-9DW trial is currently investigating the efficacy of nivolumab plus ipilimumab versus standard care (sorafenib or lenvatinib) in patients with advanced HCC naïve to systemic treatment (NCT04039707); the results are awaited.

In conclusion, immunotherapy with ICI as a monotherapy or in combination seem to be promising as a first line of treatment for patients with BCLC stage C HCC. However, the majority of trials are still ongoing and only few combinations were approved in clinical practice from regulatory authorities. Additionally, the authorization was recent in the majority of cases, so we have a very few data (or no one in some cases) regarding the phase IV, as well as real life data from the every-day clinical practice. Therefore, all reported results should still be considered with caution.

##### Second-Line Therapy: From the Standard of Care to the New Frontiers

Second-line treatments are needed for patients with good performance status, after progression or no tolerability to first-line treatment. In recent years, new advances have been made to test new systemic treatments in the second line, even if no drugs investigated in this line was tested after progression to lenvatinib. Indeed, regorafenib, the first therapeutic agent approved by the FDA in this setting, was tested in patients progressing to treatment with sorafenib (see above).

According to international guidelines [8], regorafenib, lenvatinib and ramucirumab are the biological agents used as standard of care in this setting. Shortly, in the phase III RESORCE trial, regorafenib showed to increase OS, if compared with best supportive care, from 7.8 to 10.6 months, decreasing the risk of death by 37% [10]. The CELESTIAL trial examined cabozantinib versus placebo in patients with advanced HCC who were previously treated with sorafenib [11]. Unlike the RESORCE trial, this study included also patients intolerant to sorafenib or in progression after two lines of therapy for advanced disease. In REACH-2 trial (the first phase III biomarker-driven study), ramucirumab, a human immunoglobulin G1 monoclonal anti-VEGFR2 antibody, significantly improved median OS (from 7.3 to 8.5 months) in a subgroup of patients with serum baseline alpha fetoprotein (AFP) levels ≥400 ng/mL [12].

Moving from the standard of care, immunotherapy represents a new frontiers for HCC treatment also in the second line setting. In this context, anti-PD-1 (nivolumab, pembrolizumab and camrelizumab), anti-PD-L1 (durvalumab and avelumab) and anti-CTLA-4 (tremelimumab) are being tested.

Nivolumab was the first ICI approved for patients with advanced HCC and progressed to sorafenib, based on the results of the phase I/II CheckMate-040 study [14]. In particular, the phase I part of the trial has tested escalating doses of nivolumab in 48 patients divided into three cohorts (virus-uninfected, HBV- and HCV-infected advanced HCC). The antiviral control was mandatory only in patients with HBV infection. The most frequent TRAEs were dose-unrelated and included fatigue, rash, pruritus and an increase in liver enzyme levels. Twenty-five percent of patients had a grade 3/4 TRAEs; adrenal insufficiency, diarrhea, hepatitis, and acute kidney injury were the most important. Then, the dose expansion investigated the effect of nivolumab at 3 mg/kg in 214 subjects (HCV positive, HBV positive and no viral hepatitis: 50/51/113, respectively; the last group was stratified in two subgroups: patients naïve to treatment or intolerant to sorafenib (*n* = 56) and patients progressed after sorafenib (*n* = 57)). ORR were 15% and 20% in the dose-escalation and expansion cohorts, respectively; the median OS was 15 months in the dose escalation group (95% CI = 9.6–20.2 months). The expression of PD-L1 in tumour cells was not related to response rate. The study revealed—for the first time—that nivolumab was effective and safe in patients with advanced HCC, like previously showed also in other types of cancer [74]. Notably, the trial showed that nivolumab can be safely used also in patients with HBV or HCV infections, reporting an impressive ORR with durable responses in the entire cohorts (uninfected, HBV-infected and HCV-infected patients).

Then, Pembrolizumab was approved through an accelerate process by FDA on 9 November 2018 for treatment of patients with HCC after a previous treatment with sorabenib. The KEYNOTE-224 trial [15] is a non-randomised, multicenter, open label phase II trial, which investigated the activity of pembrolizumab (200 mg every three weeks) in 104 patients affected by advanced HCC, who were refractory or intolerant to sorafenib (80% and 20% of the study population, respectively; all patients had a Child-Pugh A liver function score). The trial showed to improve the survival outcomes (median PFS: 4.9 months (95% CI 3.4–7.2); median OS: 12.9 months (95% CI 9.7–15.5); 1-year OS rate; 54% (95% CI 44–63)). Twenty-five percent of patients had grade 3-4 TRAEs and the most frequent was hypertransaminasemia (6%). Notably, none of the 26 HCV-positive (25% of the entire population) as well as none of the 22 HBV-positive patients (21% of the entire population) had worsening or re-activation of hepatitis. ORR was reported in 18 patients (17%), 77% of whom were long responders (>9 months). The trial evaluated also the relationship between PD-L1 expression and response to treatment, by using two indices of PD-L1 expression. The combined positive score (CPS: number (*n*.) of PD-L1-positive cells (both tumour and host immune cells)/ *n*. of viable tumor cells × 100) and the tumor proportion score (TPS: *n*. of PD-L1 positive tumor cells/ *n*. of viable tumor cells × 100). CPS was positive in 22 (42%) and negative in 7 (13%) patients. ORR was 25%, with the best responses in CPS and TPS positive tumours: 32% versus 20% (*p* = 0.021) and 43% vs 22% (*p*=0.088), respectively. PFS there was significantly longer in CPS positive (*p* = 0.026) but not in TPS positive patients (*p* = 0.096). In conclusion, the trial showed that pembrolizumab leads to durable responses and favorable outcomes in patients with advanced HCC who received a previous treatment with sorafenib. Then, the phase III KEYNOTE-240 randomized 413 patients affected by advanced HCC, who were refractory or intolerant to sorafenib (all patients had a Child-Pugh A liver function score), to pembrolizumab or best supportive care [75]. The trial showed a median OS of 13.9 months (95% CI: 11.6-16.0 months) for pembrolizumab versus 10.6 months (95% CI: 8.3–13.5 months) for placebo (HR: 0.781; 95% CI: 0.611–0.998; *p* = 0.0238). Median PFS for pembrolizumab was 3.0 months (95% CI: 2.8–4.1 months) versus 2.8 months (95% CI: 1.6–3.0 months; HR: 0.718; 95% CI: 0.570–0.904; *p* = 0.0022). Therefore, pembrolizumab showed a trend of better OS and PFS in this field, even if without statistical significance. However, the results were in line with the findings of KEYNOTE-224 [15]. Additionally, it is important to note that the number of patients who received an active post-study treatment was higher in the placebo arm than in the experimental arm, probably affecting the outcomes reported in the trial.

Last, the phase III KEYNOTE-394 trial (NCT03062358) is currently testing the efficacy of Pembrolizumab versus placebo in Asian pretreated patients with advanced HCC.

Camrelizumab (also known as SHR-1210) is a human IgG4 antibody against PD-1, which showed a promising activity in 58 patients with solid cancers evaluated in a phase I trial, including HCC [76]. A phase II/III trial is ongoing in China (NCT02989922), enrolling patients with advanced HCC who had failure or intolerance to prior systemic treatment. A total of 217 patients were randomized to camrelizumab (3 mg/kg every two (*n* = 109) or three weeks (*n* = 108)). Preliminary results were promising: ORR: 13.8%, 6-month OS rate: 74.7%, median time to response: 2 months, median duration of response: not reached, DCR: 44.7%, median PFS: 2.1 months. The unique TRAE reported was reactive capillary hemangioma, even if the pathogenesis, as well as the relation to the tumor response are not clear; it was observed in 66.8% of HCC patients treated. In conclusion, camrelizumab showed interesting ORR, durable response and acceptable toxicities in this Chinese trial [77].

Durvalumab and avelumab are the most relevant anti-PD-L1 agents investigated in the field of HCC. A phase I/II trial of durvalumab monotherapy in solid tumours, including HCC (*n* = 40), showed 10% ORR and median OS of 13.2 months (NCT01693562) [78]. Avelumab is a human IgG1 antibody against PD-L1; it is currently been testing as single agent, as well as in combination for advanced HCC [79]. A phase II study of avelumab, involving 30 HCC patients after sorafenib treatment, is ongoing (NCT03389126).

The anti-CTLA-4 antibody have a role in HCC treatment by increasing the expression of tumor-associated antigens, such as interleukin (IL)-1, IL-6 and macrophage inflammatory protein-1 [80,81,82].

The first anti-CTLA-4 antibody investigated in the field of HCC was tremelimumab. In particular, a phase II trial (NCT01008358) assessed the activity of tremelimumab in HCC pre-treated patients with chronic HCV infection. They received the treatment at the dosage of 15 mg/kg intravenously every 90 days until tumor progression or severe toxicity [83]. The preliminary results showed a DCR in 76.4% of patients (partial response: 17.6%) and a time to progression of 6.48 months (95% CI: 3.95–9.14 months). Notably, the trial showed that the treatment was safe also in patients with Child-Pugh stage B (42.9%).

ICIs combinations are being tested also in second-line setting for advanced HCC. The combination of nivolumab and ipilimumab for patients with advanced HCC who progressed after sorafenib treatment was firstly tested in the phase I/II CheckMate-040 study [84]. In particular, the trial randomized patients with Child-Pugh A class into three arms, according to different dosages in the combination: (a) nivolumab 1 mg/kg + ipilimumab 3 mg/kg, (b) nivolumab 3 mg/kg + ipilimumab 1 mg/kg every 3 weeks (four cycles), then nivolumab 240 mg flat dose every two weeks as maintenance, (c) nivolumab 3 mg/kg + ipilimumab 1 mg/kg every 6 weeks. The treatments were continued until disease progression or toxicity. TRAEs occurred in 37% of patients and skin toxicity-related ere the most common. However, only 5% of patients discontinued the treatment due to unacceptable toxicity. The trial demonstrated that the combination improves the ORR if compared to nivolumab monotherapy (31% versus 14%, respectively), with a promising effect on outcome (median OS: 22.8 months in the combination arm). The updated results after a minimum of 28-month follow-up, showed that 33% of patients had a response to treatment in the combination arm (8% complete response and 24% partial response) [85]. There was a long duration of response (from 4.6 to 30.5 months): maintenance of responses was recorded in 88%, 56% and 31% of patients at 6, 12 and 24 months, respectively. The ORR, as assessed by blinded independent central review using RECIST criteria modified for immunotherapy, was 35% (95% CI, 22–50%); complete and partial responses were observed in 12% and 22% of patients, respectively. Overall, the DCR was 54.0% (95% CI, 39.3–68.2%). Based on these results, in November 2019 the FDA gave a positive response about the use of nivolumab in combination with ipilimumab and in March 2020 approved the combination for patients with HCC progressed after sorafenib, according to the following schedule: nivolumab 1 mg/kg followed by ipilimumab 3 mg/kg every 3 weeks for 4 doses, followed by nivolumab 240 mg every 2 weeks or 480 mg every 4 weeks [86].

Regarding combinations between ICIs and TKI, a phase I trial (NCT02942329) investigated camrelizumab (200 mg every 2 weeks) associated to apatinib (a TKI selectively acting on VEGFR2, administrated at the dose of 125-500 mg once daily) in patients with advanced HCC, gastric or esophagogastric junction cancer [87]. The trial involved 18 patients with HCC, showing ORR of 50.0% and a median PFS of 5.8 months. The TRAEs were manageable and the discontinuation due to toxicities was reported in only one patient (grade 3 hyperbilirubinaemia). Then, a phase II study (NCT03463876) is exploring the efficacy and safety of the combination of apatinib (250 mg orally every day) and camrelizumab (200mg (3mg/kg for underweight patients) every 2 weeks) in this setting.

## 4. Future Perspectives

ICIs-based treatments welcomed new opportunities in the treatment of HCC, and not only in the advanced stage. However, despite promising results from clinical studies, only few patients benefit from ICIs [88]. Indeed, recent data showed that immunotherapies enhance survival, but their effects are limited [58]. The failure of ICI therapy might be related to the changes in the immunogenicity of cancer itself as well as of microenvironment [89,90,91]. Indeed, in this regard, the gut microbiome has gained significant attention since its alterations could affect the response to immunotherapies [92,93].

In addition, there is a lack of validated prognostic and predictive biomarkers able to guide the choice of the best treatment for each patient. In this context, some trials reported that high PD-L1 expression could be associated with poor outcome [58], even if its predictive role is still unclear and elusive, as proven by the responses to treatments both, in patients with high and low expression of PD-L1 [94]. Regarding tumor mutation burden (TMB), its role seems to be less important in HCC. In fact, HCC showed to be less immunogenic than other tumours, showing low TMB (median number of 5 Mut/Mb) [95,96,97]. Therefore, up to date, TMB is not used as potential predictive biomarker in HCC [98].

Then, other possible predictive biomarkers may be the overexpression of TIM-3 and LAG-3 in patients after receiving a previous anti-PD-1 therapy [99], whereas the epithelial-to-mesenchymal transition (EMT) could be related to resistance to immunotherapy. In fact, a study evaluating the specimens from 422 HCC patients, showed that the presence of EMTwas linked to a more aggressive disease with worst outcome [89]. Wnt/CTNNB1 mutations could also be a further biomarker of ICI resistance. Thus, the identification of better predictive biomarkers, in order to improve the efficacy of ICI therapy is a hot and challenge issue.

## 5. Conclusions

The treatment algorithm for HCC management according to BCLC stage is evolving. In this context, ICIs represent an intriguing challenge. Therefore, several clinical trials are focusing on the use of immunotherapy in HCC, alone or in combinations with TKI/antiangiogenetic agents as well as local treatment, according to the tumour stage. However, the majority of those trials are still ongoing and, until now, only a few combinations were approved in the clinical practice from the regulatory authorities. Therefore, all the reported results should be still considered with caution.

Additionally, decisions about the choice of the right sequence of treatments in HCC patients in the light of the “continuum of care” principles, is still hard. In fact, it requires careful consideration in a multidisciplinary context in order to ensure a tailored treatment for each patient.
